# Pasture and diurnal temperature are key predictors of regional Plains Spotted Skunk (*Spilogale interrupta*) distribution

**DOI:** 10.1093/jmammal/gyae063

**Published:** 2024-06-27

**Authors:** Kara M White, Amanda E Cheeseman, Joshua D Stafford, Robert C Lonsinger

**Affiliations:** Department of Natural Resource Management, South Dakota State University, 1390 College Avenue, Brookings, SD 57007, United States; Department of Natural Resource Management, South Dakota State University, 1390 College Avenue, Brookings, SD 57007, United States; U.S. Geological Survey, South Dakota Cooperative Fish and Wildlife Research Unit, Department of Natural Resource Management, South Dakota State University, 1390 College Avenue, Brookings, SD 57007, United States; U.S. Geological Survey, Oklahoma Cooperative Fish and Wildlife Research Unit, Department of Natural Resource Ecology and Management, Oklahoma State University, 007 Agricultural Hall, Stillwater, OK 74078, United States

**Keywords:** distribution, ensemble modeling, habitat suitability, Plains Spotted Skunk, predicted presence, species distribution model, *Spilogale interrupta*

## Abstract

The Plains Spotted Skunk (*Spilogale interrupta*) is a small carnivore native to central North America that has experienced significant population reductions, and there is a lack of information about the species that could inform conservation. Our study aimed to address knowledge gaps about the distribution and habitat associations of the species in South Dakota using species distribution modeling. We used species location data collected from state natural resource managers, trappers, and members of online social media groups dedicated to hunting and wildlife conservation; environmental predictors; and 6 predictive modeling algorithms (i.e., artificial neural networks, artificial classification tree analysis, generalized boosting models, maximum entropy, multivariate adaptive regression splines, and random forests) to develop climate and landcover ensemble distribution models. The most important climate and landcover predictors were mean temperature diurnal range (i.e., average monthly differences between daily high and low temperatures) and proportion of area classified as pasture. Ensemble model concordance identified approximately 31,300 km^2^ of potential Plains Spotted Skunk habitat primarily in eastern South Dakota and between the watersheds of the Missouri and James rivers. Our results offer insights that can guide conservation and inform effective management strategies for conserving Plains Spotted Skunk populations in the northern Great Plains. The promotion of low-intensity agricultural practices such as maintaining pastures, farm buildings, fences rows, and the management of woodland encroachment may improve habitat suitability and facilitate the recovery of plains spotted skunks in the region.

## Teaser

We mapped Plains Spotted Skunk distribution using ensemble modeling techniques. Our results indicate ~31,000 km² of suitable habitat, primarily in eastern South Dakota. Management efforts can focus on promoting sustainable farming practices and managing woodlands to facilitate species conservation in the region.

Anthropogenic disturbances including land-use change, disruption to biogeochemical cycles, and climate change have caused declines in species abundance and distribution (Wilson et al. 2016). Effective conservation and recovery programs are essential for protecting imperiled wildlife populations and their habitats (Tilman et al. 2017). Observed extinction rates have surpassed natural background rates, and the loss of species poses a severe threat to ecosystem stability and functioning, impacting key processes such as nutrient cycling, climate regulation, and maintenance of genetic diversity (Pimm et al. 2014). Therefore, evidence-based management practices are necessary to achieve conservation goals.

Predictions about the spatial distribution of species are important components of conservation management (Marmion et al. 2009), but generating reliable spatial predictions is challenging for species that occur at low densities and are often cryptic, nocturnal, and solitary (Wilson and Delahay 2001; Balme et al. 2009). To overcome these challenges, species distribution modeling offers an appealing approach because of its broad applicability and limited data requirements, making the approach particularly well-suited for sparse data sets (Aubry et al. 2017). Species distribution models estimate the probability of species occurrence across geographic space by using presence-only data sets that are matched with environmental and climatic characteristics (Elith et al. 2006). Importantly, species distribution models can account for instances where a target species is present but not observed (i.e., falsely absent). Conservation managers widely employ species distribution models to understand how environmental attributes and anthropogenic land use relate to species presence. Moreover, species distribution models aid in identifying critical threats and prioritizing specific management actions and strategies such as habitat protection and restoration initiatives (Sofaer et al. 2019).

The Plains Spotted Skunk (*Spilogale interrupta*) is a small (i.e., ≤1.1 kg) carnivore native to central North America with a historical distribution spanning from Manitoba, Canada to Tamaulipas, Mexico, and from the Mississippi River to the Rocky Mountains (McDonough et al. 2022). Long-term harvest trends of spotted skunks east of the Rocky Mountains, i.e., encompassing plains spotted skunks and eastern spotted skunks (*S. putorius*), indicated a precipitous population decline that commenced in the 1940s (Gompper and Hackett 2005). The exact causes of the reduction remain unknown; however, several hypotheses suggest that intensified land-use practices, such as agricultural expansion reducing habitat, widespread use of agricultural pesticides decreasing prey abundances, and alterations in predator communities increasing competition, may play significant roles (Gompper and Hackett 2005; Gompper 2017).

Perceived population declines led to a petition to consider plains spotted skunks under the U.S. Endangered Species Act, prompting the U.S. Fish and Wildlife Service (USFWS) to conduct a species status assessment (U.S. Fish and Wildlife Service [USWFS] 2012). After thorough evaluation, the USFWS ruled that listing protection for the species was not warranted but acknowledged the species as data-deficient (USFWS 2023). Few studies have investigated plains spotted skunks, and even fewer have focused on the northern portion of the species range (Crabb 1948; Polder 1968). As a result, much of our knowledge about plains spotted skunks has been inferred from studies conducted in the southeastern periphery of the species range (Lesmeister et al. 2010, 2013; LaRose et al. 2022; Perkins et al. 2022). Further insights were largely limited to those deduced from studies of closely related species (Gompper 2017).

Previous studies on the distribution of plains spotted skunks have predominantly focused on southern portions of the species range (LaRose et al. 2022; Perkins et al. 2022) or have not differentiated between plains and eastern spotted skunks (Cheeseman et al. 2021a). Importantly, our study area was situated well outside any historical or potential contact zones between the 2 species, ensuring our findings are directly applicable to the Plains Spotted Skunk. Previous studies also relied on a single modeling algorithm, which employed a maximum entropy approach, to assess habitat suitability. The reliance on a single algorithm (e.g., maximum entropy) has inherent limitations and predictions are subject to the assumptions and constraints of that model. Therefore, a singular modeling approach may fail to capture the full complexity of habitat suitability, and consideration of competing modeling algorithms may yield varying predictions under the same conditions (Araújo and New 2007). To this end, ensemble modeling has demonstrated its effectiveness in reducing errors and improving generalization by capturing the overall trend of the data while minimizing the effects of individual model biases or limitations and is useful when the data are noisy, or the model is subject to overfitting or uncertainty (Marmion et al. 2009). Rather than relying on a single approach, we combined multiple algorithms using an ensemble technique to improve our understanding. Furthermore, climate variables encompass long-term climatic conditions, which directly affect physiological tolerances and life history traits of a species, whereas landcover variables capture characteristics of the physical landscape such as vegetation type, habitat structure, and anthropogenic modifications. Recognizing that climate and landcover variables were distinct aspects of the environment that could affect species distributions, we chose to model climate and landcover variables separately to better understand their individual effects on the distribution of plains spotted skunks.

In our study, we differentiate between distribution and suitability to reflect different aspects of Plains Spotted Skunk ecology. We use the term distribution to describe Plains Spotted Skunk occurrences across the landscape, providing insights into current and historical presences of the species. In contrast, we use the term suitability to denote areas that possess habitat characteristics conducive to supporting life processes of the species (Hirzel et al. 2006). However, we recognize that suitability does not necessarily equate to current occupancy, as it may not account for factors such as historical extirpation or interspecific competition that may preclude presence of a species in otherwise suitable environments (Guisan et al. 2017). Thus, our study objectives encompassed 2 main aspects: (1) identifying climate and landcover factors associated with the distribution of plains spotted skunks; and (2) modeling habitat suitability to identify priority areas for targeted management efforts (e.g., habitat protection and augmentation) in South Dakota, United States. Drawing on previously inferred habitat associations (Perkins 2017; Cheeseman et al. 2021a; LaRose et al. 2022), we predicted that the distribution of plains spotted skunks in South Dakota would be positively associated with measures of temperature and precipitation as well as with tree and shrub cover, and negatively associated with crop cover.

## Materials and methods

### Study area

South Dakota, located in the northern Great Plains, covers ~197,730 km^2^ (USDA National Agricultural Statistics Service 2017). The Missouri River bisects the state and delineates the transitional zone between the semiarid west and moist eastern region. The western region consists of rolling plains, buttes, and canyons, whereas the east consists of nearly level land with many small lakes and ponds (Barker and Whitman 1988). Temperate grasslands characterize most of the state. Trees and shrubs are restricted and clustered near rivers and in shelterbelts with the exception of the Black Hills in the west, which supports coniferous forest stands (Barker and Whitman 1988). South Dakota is largely rural, with an average of 4.3 people/km^2^ (US Census Bureau 2020). Land is primarily used for agriculture (~85%; USDA National Agricultural Statistics Service 2017) with croplands in the east and livestock grazing in the west where higher temperatures, lower humidity, and lower precipitation limit crop growth. Average precipitation, occurring primarily from April through September, increases from northwest to southeast and ranges from 38 to 71 cm annually. Large temperature extremes are common. The lowest temperature is −50 °C, although winter daytime high temperatures average 0 °C. The highest temperature is 49 °C, although summer daytime high temperatures average 32 °C (Frankson et al. 2022).

### Location data

We conducted a widespread campaign to solicit Plains Spotted Skunk observations in South Dakota. We encouraged respondents to report Plains Spotted Skunk observations via phone, email, or online through an iNaturalist project page that we created to aggregate online reports. Targeted respondents included state natural resource managers, academic institutions, furbearer license holders and state trapper association members, agricultural landowners, and members of various hunting and wildlife-oriented social groups. Information about our campaign appeared in multiple news publications throughout the state including in print, online, and over the radio. We also queried the VertNet (VertNet 2016) and Global Biodiversity Information Facility (GBIF.org 2021) databases, reviewed published (Fino et al. 2019) and ongoing wildlife research in South Dakota, and accessed data maintained by the South Dakota Game Fish and Parks Natural Heritage Program. Finally, we reviewed information from the South Dakota Department of Transportation’s roadkill image database for all roadkill tagged as a “small animal” (South Dakota Department of Transportation 2020).

We acknowledge the potential for observational bias toward habitats linked to human activity in our data compilation process. For contemporary crowd-sourced data, we took specific steps to minimize the impact of this bias. We excluded Plains Spotted Skunk observations that either lacked requisite spatial information or failed to meet our data quality criteria (i.e., reports without photographs, video, or a detailed description of the Plains Spotted Skunk; Perkins et al. 2022). Additionally, we reviewed records from state and museum databases; this process involved checking for taxonomic accuracy, removing duplicate records, and filtering out entries that lacked essential spatial information. We georeferenced all remaining records and estimated their locational uncertainty to be within a section (~2.6 km^2^) based on plat maps of marked locations provided by trappers. Using the “spThin” package in the R programming environment version 4.2.2 (Aiello-Lammens et al. 2019; R Core Team 2023), we refined our data set by systematically omitting one of each pair of records that were located within 3 km of each other, which was slightly larger than our estimated geographic uncertainty for each spotted skunk location. Our process aimed to prevent potential duplication in the data set, reduce the effects of spatial autocorrelation, and mitigate the impact of observational bias associated with habitats linked to human activity, ensuring that each location in our analysis represented a distinct observation site. Importantly, for the purpose of our analysis, we restricted the data to records from 1985 onward, ensuring that we focused on more recent data and to avoid overestimating the putative distribution of the species by including older records. Our decision was informed by the relative stability and minimal change of land use in South Dakota between the years 1982 to 2017 (USDA 2017).

Although species distribution models require presence-only data sets, Elith et al. (2006) found that presence–absence models performed better than presence-only models. Due to the inherent challenge of determining true absences, pseudo-absences are commonly generated as substitutes (Zarnetske et al. 2007). Research radio-tracking plains spotted skunks in South Dakota during 2021 to 2022 documented movements up to ~18 km (White et al. 2022). In order to establish our modeling domain, we buffered Plains Spotted Skunk locations by a 20-km radius, slightly exceeding the greatest observed relocation distance for a radio-collared individual. Following the recommendations of Barbet-Massin et al. (2012), we randomly generated an equal number of pseudo-absences to presence points to create a final data set.

### Environmental data

We obtained rasters for elevation and 19 bioclimatic variables (Table 1) measured at baseline conditions from 1970 to 2000 and averaged (WorldClim version 2.1; Fick and Hijmans 2017). We chose a resolution of 2.5 arc minutes (~3 km^2^), which closely matched the georeferenced locational uncertainty of the Plains Spotted Skunk data set. We matched all presence and pseudo-absence points to each raster and extracted climate data at each point. We obtained an existing vegetation cover raster at a resolution of 30 m^2^ from 2001 LANDFIRE (https://landfire.cr.usgs.gov/). We opted to use vegetation cover data from 2001 as it represented an approximate temporal midpoint in our Plains Spotted Skunk observation period, thereby minimizing the potential effect of landcover changes over time in our analysis. We reclassified existing vegetation cover types into broader categories of forest, shrub, herbaceous, developed vegetation, and crop cover (Table 2). For purposes of interpretation, we considered all landcover variables to be subject to some degree of anthropogenic influence. Before matching and extracting landcover data for all presence and pseudo-absence locations, we used the “landscapemetrics” package in R (Hesselbarth 2022; R Core Team 2023) to calculate area, aggregation (i.e., number of patches and percent like adjacencies), and landcover diversity indices (i.e., Shannon’s diversity index, Shannon’s evenness index, Simpson’s diversity index, and Simpson’s evenness index) for each reclassified existing vegetation cover type at a resolution of 3 km^2^ (Table 2). After assessing collinearity among predictor variables using Spearman’s rank correlation coefficient, we used variable importance of projection (VIP) scores to identify the most important climate and environmental variables. In cases where 2 predictors were strongly correlated (i.e., |*r*| > 0.7; Dormann et al. 2013), we retained the predictor with the higher VIP score.

**Table 1.** Bioclimatic and landscape predictors considered for climate-based species distribution modeling of plains spotted skunks (*Spilogale interrupta*) in South Dakota. Bioclimatic and elevation predictors were collected from the Worldclim database (www.worldclim.com) and averaged baseline conditions from 1970 to 2000 at a resolution of 2.5 arc minutes (~3 km^2^), whereas elevation was collected at a resolution of 30 m^2^. Predictors in the final set used for modeling are denoted with an asterisk.

Type	Predictor descriptions
Bioclimatic (temperature)	Annual mean temperature
	Mean diurnal range (mean of monthly (max temp - min temp))*
	Isothermality (mean diurnal range/temperature annual range) (×100)
	Temperature seasonality (standard deviation × 100)
	Maximum temperature of warmest month
	Minimum temperature of coldest month*
	Temperature annual range
	Mean temperature of wettest quarter*
	Mean temperature of driest quarter
	Mean temperature of warmest quarter
	Mean temperature of coldest quarter
Bioclimatic (precipitation)	Annual precipitation
	Precipitation of wettest month
	Precipitation of driest month
	Precipitation seasonality (coefficient of variation)*
	Precipitation of wettest quarter
	Precipitation of driest quarter
	Precipitation of warmest quarter*
	Precipitation of coldest quarter
Landscape	Elevation

**Table 2.** Initial predictors considered for Plains Spotted Skunk (*Spilogale interrupta*) landcover distribution models; landcover variables were collected at a resolution of 30 m^2^ from 2001 LANDFIRE data (www.landfire.gov). Predictors in the final set used for modeling are denoted with an asterisk.

Type	Predictor	Description
Area	Water	Open water
	Developed vegetation*	Natural vegetation developed due to human activities; e.g., roadsides, section breaks between fields
	Developed open space	Mixture of constructed materials and vegetation; impervious surfaces < 20% of total cover; e.g., parks, lawns
	Developed low intensity	Mixture of constructed materials and vegetation; 20% < impervious surfaces < 49% of total cover
	Developed medium intensity	Mixture of constructed materials and vegetation; 50% < impervious cover < 79% of total cover
	Developed high intensity*	Impervious surfaces that account for 80% to 100% of total cover
	Roads	Roads
	Barren	Bedrock, glacial debris, talus, slides, quarries, gravel pits, and other accumulations of earthen material
	Crop cover*	Areas used to produce annual row crops
	Pasture	Areas used for livestock grazing or to produce hay crops
	Herbaceous wetlands	Herbaceous vegetation where substrate is periodically saturated or covered with water
	Sparse vegetation	Sparse vegetation canopy < 10% of total cover
	Tree cover*	Trees > 5 m tall, including forest and shelterbelts
	Shrub cover*	Shrubs and young trees < 5 m tall
	Herbaceous cover*	Graminoid or forb vegetation
Aggregation	Number of patches*	Number of patches
	PLAJ	Percent of like adjacencies
Diversity	SHDI	Shannon’s diversity index
	SHEI	Shannon’s evenness index
	SIDI*	Simpson’s diversity index
	SIEI	Simpson’s evenness index

### Species distribution models

To predict Plains Spotted Skunk distribution based separately on climate and landcover, we fitted location data using 6 predictive modeling algorithms that performed well in comparative studies (Elith et al. 2006): artificial neural networks (ANN); classification tree analysis (CTA); generalized boosting models (GBM); maximum entropy (MAXENT); multivariate adaptive regression splines (MARS); and random forests (RF). We fitted models with the “biomod2” package in R (R Core Team 2023; Thuiller et al. 2023) using default settings. To calibrate the models, we randomly selected 70% of the locations and trained the models. We then evaluated trained models against the 30% of locations that were not included in the calibration (Thuiller et al. 2019). We performed 10 runs for each of the 6 modeling algorithms (60 total model runs; Barbet-Massin et al. 2012) for both climate and landcover models. We calculated the area under the curve (AUC) of the receiver operating characteristic (ROC) and the true skills statistics (TSS) to assess model accuracy. The ROC is most commonly reported in distribution studies (Lobo et al. 2007) and is generated by testing all conceivable thresholds to classify scores into confusion matrices, calculating sensitivity and specificity for each matrix, and then plotting values with sensitivity on 1 axis and the proportion of false positives (i.e., 1 − specificity) on the other (Fielding and Bell 1997). More recently, TSS has been recommended as an alternative measure of model performance (Allouche et al. 2006), and is similar to Cohen’s kappa but is independent of prevalence, and its results have been significantly correlated to the ROC metric (Allouche et al. 2006). Values of ROC range from 0 to 1 with values > 0.5 indicating model performance better than random (Fielding and Bell 1997), whereas TSS values range from −1 to 1 with values > 0 indicating model performance better than random (Allouche et al. 2006). We calculated sensitivity (the proportion of correctly classified presences) and specificity (proportion of correctly classified absences) to quantity omission and commission errors, respectively (Fielding and Bell 1997). To address potential variability in results across modeling techniques, we employed an ensemble model approach to combine models with TSS values > 0.7 using a weighted average method (Marmion et al. 2009). To transform continuous predictions into binary predictions, we used the threshold that maximized the TSS. We used ArcGIS version 10.7.1 (ESRI 2019) to map ensemble predictions, and zonal statistics in the Spatial Analyst toolbox to quantify areas of suitable habitat. To evaluate the importance of predictor variables, we randomly permuted each variable 3 times and created 3 new data sets for each permutation (Anderson 2001). We trained models using the resulting data sets with the same specifications as the original models, compared model predictions for each permutated data set to the original data set, and ranked predictor variables based on the magnitude of differences (Wei et al. 2015).

## Results

### Location data

We collected a total of 185 Plains Spotted Skunk records from 46 counties across South Dakota from 1954 to 2022. Most reports were provided by trappers (28.6%); followed by the South Dakota Game, Fish, and Parks Natural Heritage database (22.7%); hunting and wildlife social groups (20.0%); and South Dakota State University research projects (16.2%). Other sources included museum databases (5.9%), agricultural landowners (2.2%), natural resource managers (1.6%), and miscellaneous sources (2.7%). We retained 129 records (i.e., 23 records from or before 1999 and 106 records from 2000 and onward) after thinning the data set to match the georeferenced location uncertainty and availability of contemporary predictor data.

### Climate ensemble distribution models

**Fig. 1.**
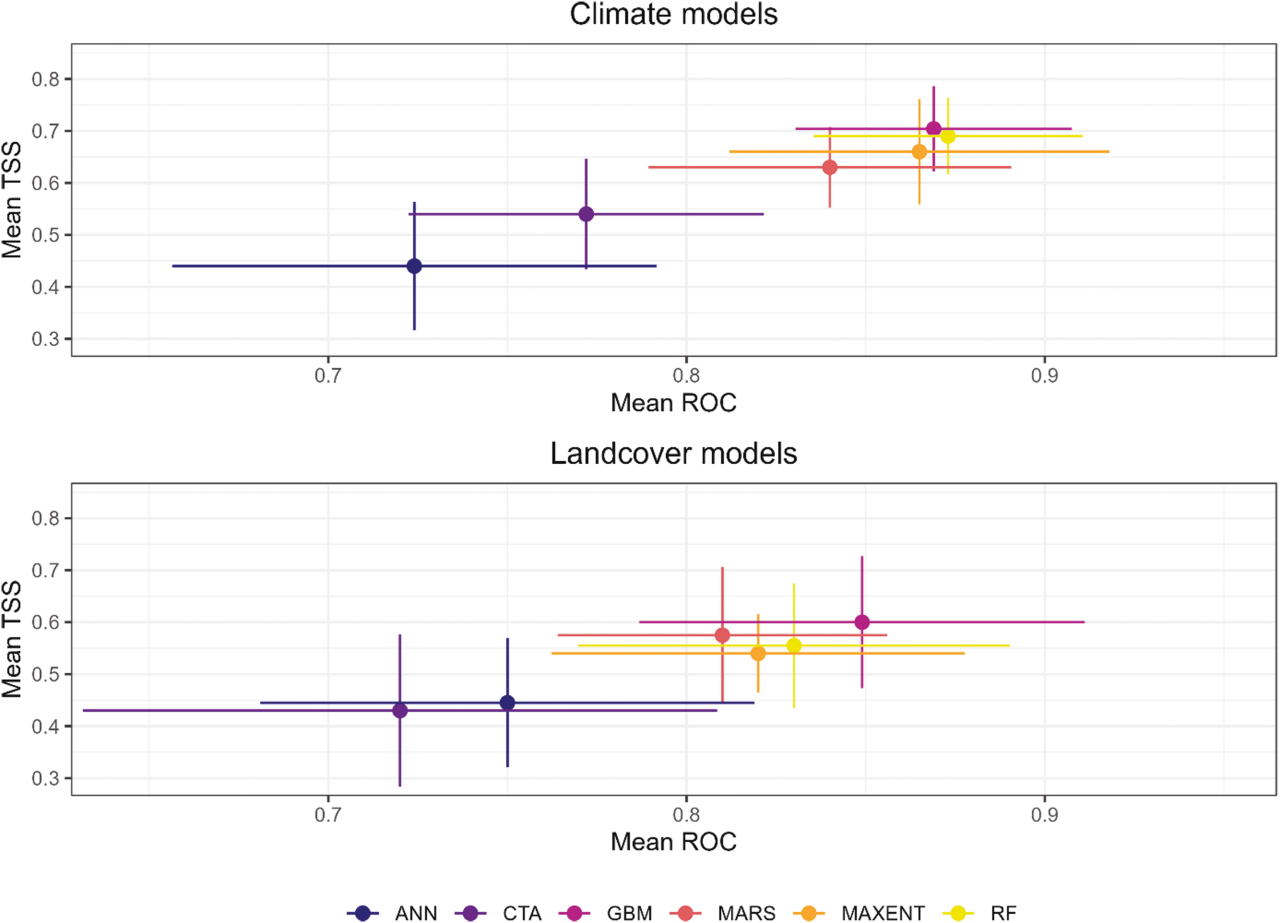
Model performance comparison of 6 modeling techniques: artificial neural networks (ANN); classification tree analysis (CTA); generalized boosting models (GBM); multivariate adaptive regression splines (MARS); maximum entropy (MAXENT); and random forests (RF) by area under the receiver operating curve (ROC) and true skill statistic (TSS) values for 10 model iterations based on climate (top) and landcover predictors (bottom) for Plains Spotted Skunk (*Spilogale interrupta*) predicted distribution in South Dakota. Points represent mean estimates and solid lines represent standard deviations.

**Fig. 2.**
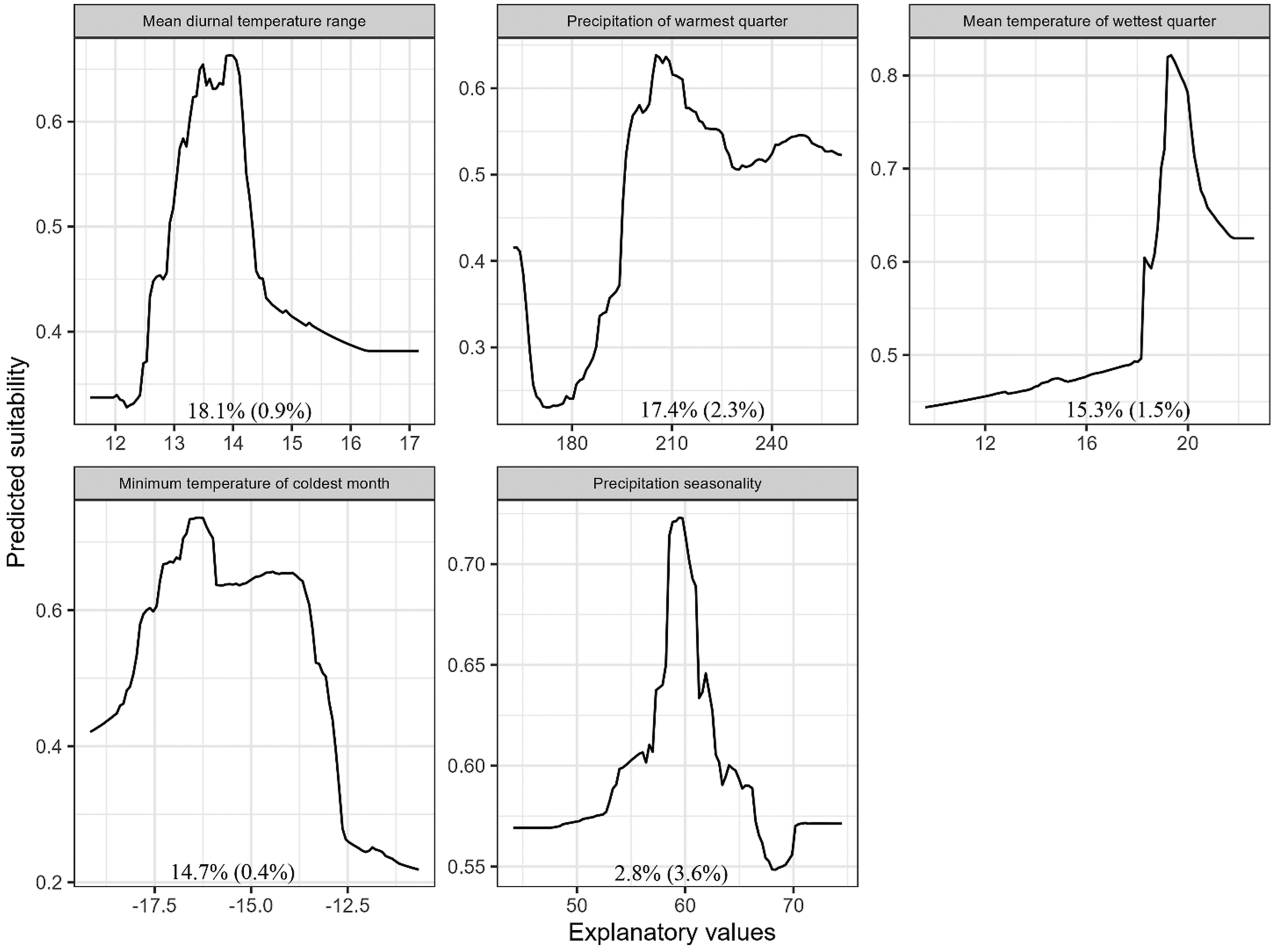
Predicted response curves for temperature (°C) and precipitation (mm) variables used in the ensemble model of climate suitability for plains spotted skunks (*Spilogale interrupta*) in South Dakota, United States; values presented within each plot represent the mean percent importance (± SD) of predictors from 3 permutations used in ensemble models.

**Fig. 3.**
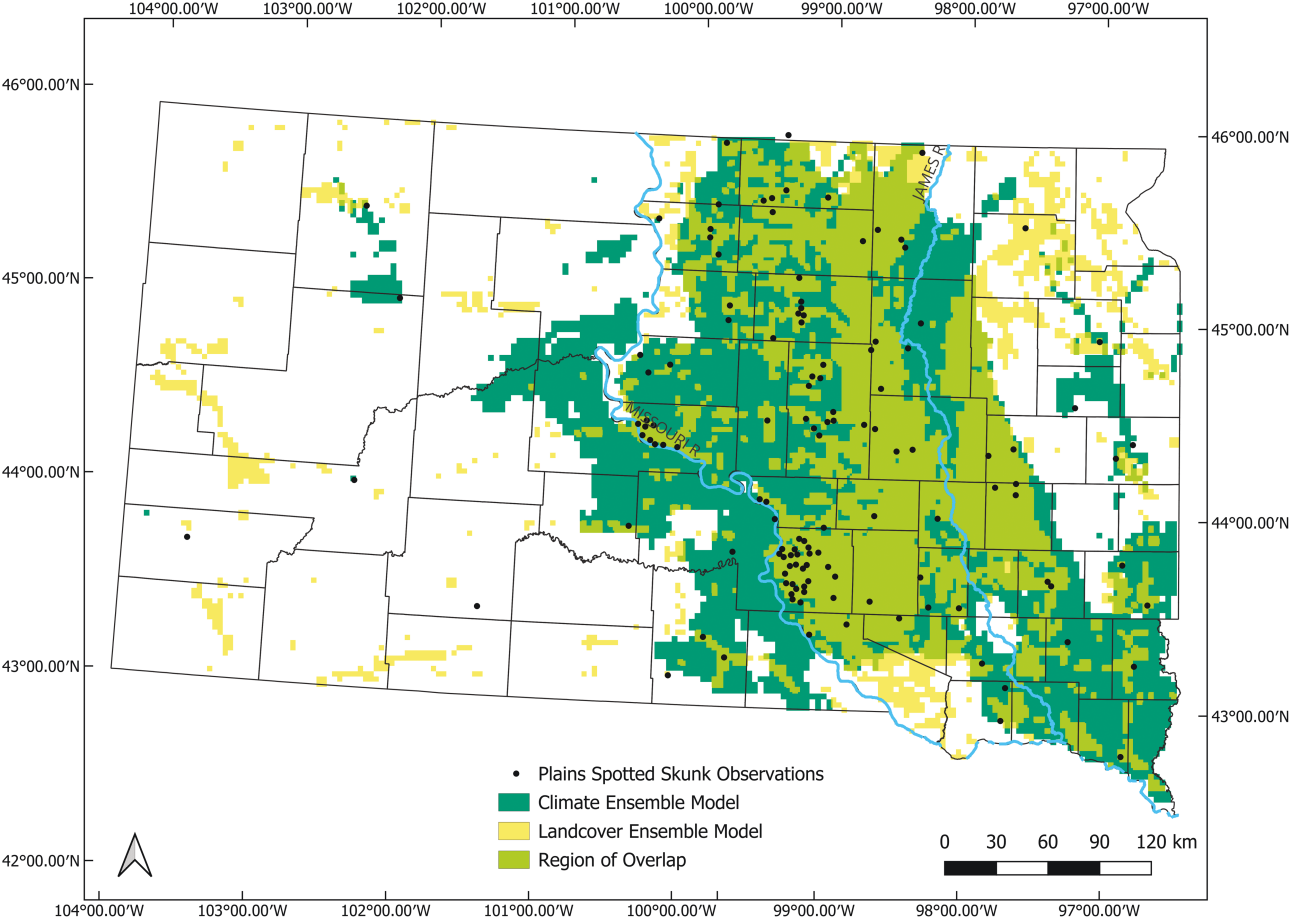
Predicted landscape suitability for plains spotted skunks (*Spilogale interrupta*) in South Dakota, United States based on climate ensemble model predictions and landcover ensemble model predictions; points represent only the spotted skunk observations used in the models to assess suitability.

Based on 10 runs for each of the 6 modeling algorithms, mean ROC ranged from 0.72 (ANN) to 0.87 (RF) indicating good to excellent predictive power, whereas mean TSS ranged from 0.44 (ANN) to 0.70 (GBM) indicating moderate to substantial agreement (Fig. 1; Swets 1998). Ensemble model performance was classified as excellent based on the ensemble weighted mean AUC value (0.97) with substantial agreement based on the ensemble weighted mean TSS value (0.83). The ensemble model correctly classified 93.0% of true presences (i.e., sensitivity) and 89.9% of true absences (i.e., specificity). The most important climate predictor of Plains Spotted Skunk distribution was mean diurnal temperature range, followed by precipitation of warmest quarter, mean temperature of wettest quarter, minimum temperature of coldest month, and precipitation seasonality (Fig. 2). Higher predicted suitability was observed at lower values of mean diurnal temperature range (Fig. 2). For precipitation of the warmest quarter, higher predicted suitability was associated with greater precipitation values. Additionally, predicted suitability generally increased as the mean temperature of the wettest quarter increased before peaking and decreasing at the greatest values. Predicted suitability increased with higher values of minimum temperature of the coldest month, and then stabilized between values of −15.8 and −13.8 °C before decreasing over less commonly observed extreme values. In terms of precipitation seasonality, intermediate values were associated with the greatest predicted suitability. The climate ensemble model predicted ~75,564 km^2^ of habitat for plains spotted skunks, primarily in eastern South Dakota between the Missouri River and James River watersheds (Fig. 3).

### Landcover ensemble distribution models

**Fig. 4.**
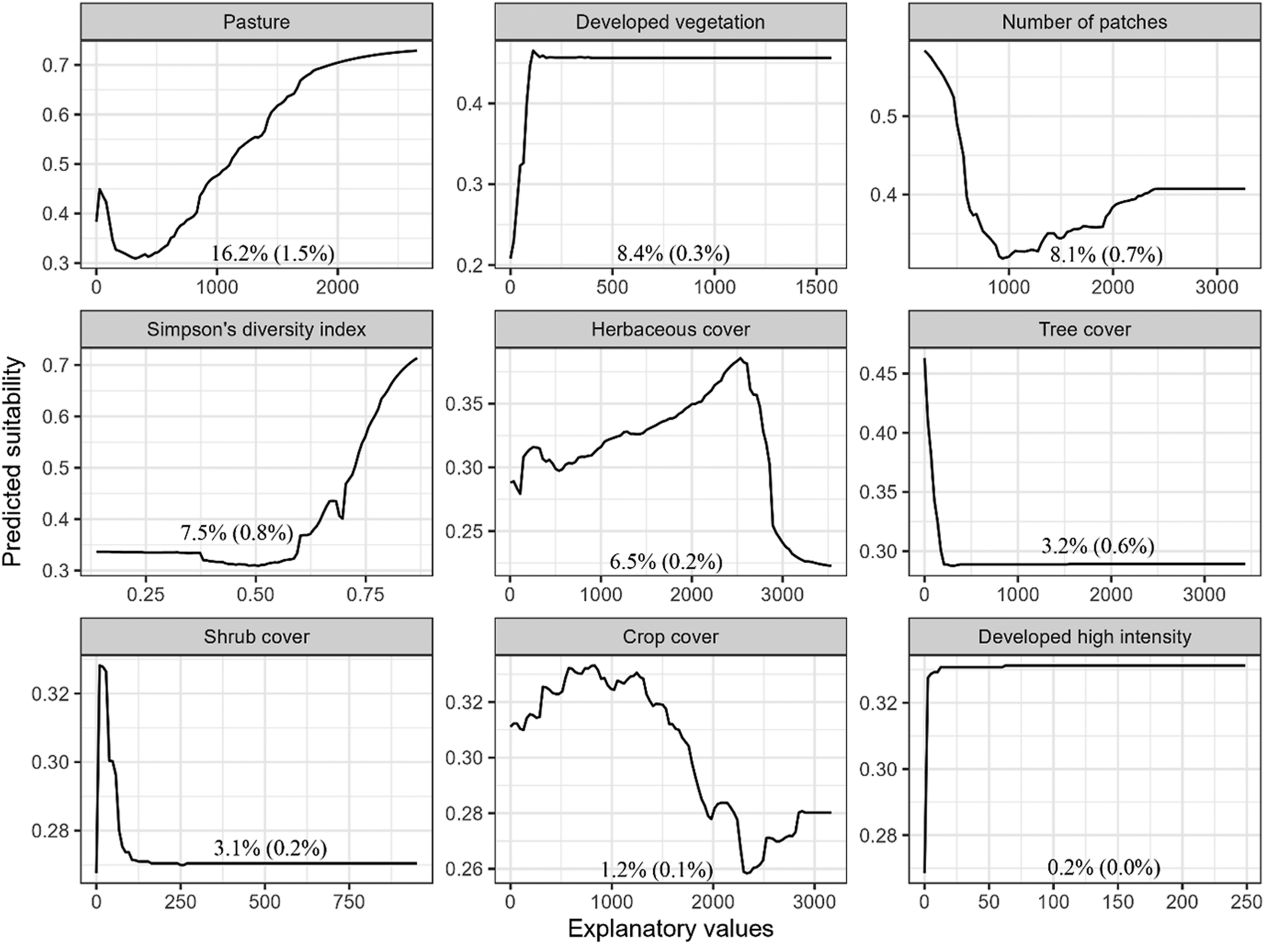
Predicted response curves for variables (measured in m^2^) used in the ensemble model of landcover suitability for plains spotted skunks (*Spilogale interrupta*) in South Dakota, United States; values presented within each plot represent the mean percent importance (± SD) of predictors from 3 permutations used in ensemble models.

Based on 10 runs for each of the 6 modeling algorithms, mean ROC values ranged from 0.72 (CTA) to 0.85 (GBM) indicating good to excellent predictive powers (Fig. 1; Swets 1998). Average TSS values ranged from 0.43 (CTA) to 0.60 (GBM) indicating moderate agreement. Ensemble model performance was classified as excellent based on the ensemble weighted mean AUC value (0.97) with substantial agreement based on the ensemble weighted mean TSS value (0.83). The ensemble model correctly classified 83.7% of true presences (i.e., sensitivity) and 99.2% of true absences (i.e., specificity) in the data set. The most important landcover predictor of Plains Spotted Skunk distribution was pasture, followed by developed vegetation, number of patches, Simpson’s diversity index, and herbaceous cover; the remaining landcover predictors had permutation importance values < 5% (Fig. 4). Pasture was positively associated with predicted suitability (Fig. 4), which increased as values of developed vegetation increased before reaching an asymptote. At smaller values, the number of patches was negatively associated with predicted suitability, but a positive trend was observed as values of number of patches were >1,000. Landscape diversity showed a positive association with predicted suitability. Although the model demonstrated a strong negative association between predicted suitability and herbaceous cover at the highest cover values, predicted suitability remained relatively consistent across most values of herbaceous cover. The landcover ensemble model predicted ~42,174 km^2^ of potential habitat for plains spotted skunks, primarily in eastern South Dakota between the Missouri River and James River watersheds (Fig. 3), of which ~31,302 km^2^ overlapped with habitat predictions based on the climate ensemble model.

## Discussion

Examining the relationship between shifting climate patterns and land-use patterns is essential for assessing the effects of human activities on species conservation and designing effective management strategies. Our findings were consistent with research indicating that minimum temperature of the coldest month was an important predictor of Plains Spotted Skunk habitat suitability at large spatial scales and supported the hypothesis that cold temperatures constrained the distribution of Plains and Eastern Spotted Skunk (Cheeseman et al. 2021a). Spotted skunks remain active throughout winter (Kinlaw 1995) and thermoregulation during cold temperatures may be energetically costly. Furthermore, our findings revealed the sensitivity of Plains Spotted Skunk distribution to extreme temperature fluctuations, with such fluctuations identified as the most important predictor, particularly at colder extremes. Our results provide support for the hypothesis that abiotic conditions at higher latitudes may limit species distribution (Darwin 1859). Extreme weather events have become more frequent (Gallant et al. 2014), and researchers have attributed Arctic warming to the destabilization of both the jet stream and the polar vortex, which pushes unseasonably cold temperature extremes into temperate latitudes (Hanna et al. 2016). Our findings indicate that while winter temperatures are generally increasing, climate destabilization may have negative effects on plains spotted skunks.

Though measures of precipitation were identified as less important than measures of temperature in our ensemble model, they may still be important climatic drivers of Plains Spotted Skunk distribution. Habitat suitability for plains spotted skunks has been positively associated with precipitation at broad (i.e., 10-km resolution across the entire geographic range; Cheeseman et al. 2021a) and local (i.e., at the state level using a 4-km resolution; Perkins 2017) scales, and our results in South Dakota aligned with previous patterns and our hypotheses. Precipitation drives ecosystem functioning in semiarid and arid grasslands, influencing not only primary production but community composition and abundance of species at higher tropic levels (Parmenter et al. 1999; Barnett and Facey 2016). It is likely that the observed relationship between plains spotted skunks and precipitation is related to prey availability, which has been previously proposed as an important driver of Plains Spotted Skunk populations (Cheeseman et al. 2021b). Additionally, precipitation patterns are important drivers of agricultural practices, influencing decisions about agricultural activities and irrigation strategies. Our climate ensemble model primarily identified areas east of the Missouri River as suitable Plains Spotted Skunk habitat in South Dakota, where considerably more agricultural activity occurred, and more precipitation fell annually than in western parts of the state.

Historical agricultural practices across the Great Plains were characterized by small farmsteads, which promoted Plains Spotted Skunk occurrence by aggregating prey resources and providing cover (Choate et al. 1973). Pasture, developed vegetation (i.e., areas of natural vegetation developed due to human activities), and areas of herbaceous cover were important landcover predictors of Plains Spotted Skunk distribution in South Dakota, a pattern consistent with positive associations documented between plains spotted skunks and pasture and developed vegetation (Crabb 1948; Choate et al. 1973). In contrast, pasture and herbaceous cover were not identified as important for Plains Spotted Skunk in the southern Great Plains and developed vegetation was not considered (Perkins 2017). Contrasting patterns may be attributed to differences in the scale of our study or due to regional differences in management practices, species interactions, and habitat characteristics between the southern and northern Great Plains and demonstrate the need to consider regional differences when assessing habitat suitability for species of concern. Our results indicated that Plains Spotted Skunk habitat suitability was negatively associated with crop cover (although the importance from our model was low; i.e., <5%), supporting the hypothesis that increased agricultural intensity may have contributed to spotted skunk population declines (Gompper and Hackett 2005). Our results were consistent with Plains and Eastern Spotted Skunk distributional work although negative associations between crop cover and habitat suitability were more pronounced at broader scales (Cheeseman et al. 2021a). One possible explanation is that at broader scales agricultural practices can cause greater landscape homogenization, which can lead to smaller areas of habitat and fewer patches of remaining habitat. Furthermore, row-crop agriculture may be associated with lower invertebrate diversity (Burcher et al. 2007), a primary food source of plains spotted skunks (Crabb 1948; Kinlaw 1995), and may be a contributing factor to relatively low Plains Spotted Skunk habitat suitability in crop cover. To support conservation efforts for plain spotted skunks in agricultural settings, it may be beneficial to implement management strategies that promote low-intensity agricultural practices such as maintaining areas of pasture, and preserve features such as fence rows, farm buildings, and crop storage, which provide prey and protection from predators.

The distribution and abundance of predator communities are also influenced by landcover and land-use patterns within an ecosystem (Prugh et al. 2009). Our ensemble model response to landscape diversity was consistent with research on Plains and Eastern Spotted Skunk habitat suitability at broader scales that indicated a positive relationship between landscape diversity and habitat suitability (Cheeseman et al. 2021a). Previous research found a positive association between landscape diversity and mesopredator abundance (Prugh et al. 2009). However, the rise of large-scale farming has resulted in a decrease in landscape diversity, which has been associated with the decline of spotted skunk populations (Gompper and Hackett 2005). Based on our findings, management efforts may benefit from promoting landscape diversity through the creation of heterogeneous habitats and preserving patches of remaining habitat. Furthermore, prioritizing management efforts for the species in the region may involve promoting more diverse land-use practices while acknowledging the challenges associated with large-scale farming.

However, the relationship between habitat suitability and the number of patches is likely scale-dependent (Riitters et al. 1997). The positive association that we observed between predicted suitability and the number of patches (for values > 1,000) was consistent with patterns reported at a broad scale (although at a broader scale this variable was of least importance; Cheeseman et al. 2021a). The number of patches is a relatively simple metric that may not best describe complex patterns of fragmentation that result from anthropogenic land-use practices. At small spatial scales, landscapes may be more homogeneous (i.e., fewer patches) making it difficult to discern a pattern. Furthermore, the number of patches may not be a good indicator of habitat quality if landscape patches are comprised of lower-quality habitats. Rather, the quality and diversity within individual patches may be more important for determining Plains Spotted Skunk habitat suitability at smaller scales.

While the remaining predictors demonstrated lesser importance in predicting habitat suitability (i.e., <5%), previous studies on the distribution of plains spotted skunks have identified the importance of tree and shrub cover (Perkins 2017; LaRose et al. 2022). For example, research conducted in Arkansas and Missouri found a positive association between predicted Plains Spotted Skunk habitat suitability and the presence of tree and shrub cover (Lesmeister et al. 2013; LaRose et al. 2022). We hypothesized that we would find a similar result; however, our hypothesis was not supported by our findings which revealed a strong negative association between Plains Spotted Skunk predicted habitat suitability and tree cover. In South Dakota, woodlands are typically sparse and primarily found as windbreaks or shelterbelts (Barker and Whitman 1988), although fire suppression and other land-use practices have promoted woodland encroachment in some areas (e.g., *Juniperus* spp.; Kaskie et al. 2019). Trees provide perching sites in agricultural settings and may lead to increased concentrations of raptors (Machar et al. 2017) and predation risk for plains spotted skunks (Lesmeister et al. 2010). Conversely, while predicted suitability generally remained low across all values of shrub cover, we did not observe as strong of a negative association as we did with tree cover. Rather, predicted habitat suitability was highest when shrub cover was relatively low. Research indicates that the dense composition of early successional woodland communities, including shrubs, can enhance prey abundance and reduce predator search efficiencies (Loggins et al. 2019). Consequently, the impact of woodland encroachment on the distribution of plains spotted skunks may depend on the stage of woodland development. In early successional stages, shrubby woodland encroachment may create favorable conditions for plains spotted skunks, providing prey-rich foraging opportunities while also offering protective cover. In mature woodlands, the concentration of raptors may pose a higher risk to plains spotted skunks, hindering survival, and decreasing habitat suitability. If the negative association we observed between trees and predicted suitability for plains spotted skunks was due to avian predation, efforts to reduce woodland encroachment and establishment may mitigate (or reduce) avian predation and facilitate conservation efforts for plains spotted skunks.

In conclusion, our study used ensemble species distribution modeling to investigate the relationship between climate patterns, landcover characteristics, and the distribution of plains spotted skunks in South Dakota. By focusing on understudied areas within their range, our research highlights the need for habitat assessment at local scales and the limited utility of extrapolations from disparate ecoregions. We acknowledge that while large-scale patterns provide valuable insights into species ecology, they may not capture the nuances of local factors influencing distribution.

Our research offers actionable insights for conservation efforts by identifying areas with the greatest potential for success in supporting Plains Spotted Skunk populations. Furthermore, our findings serve as a guide for future surveys by pinpointing areas of high suitability that lack current species presence data, thereby efficiently directing survey efforts to areas most likely to yield new insights into Plains Spotted Skunk ecology. Our models integrate observations over a 37-year period, depicting an average scenario of habitat suitability for the Plains Spotted Skunk. It is important to note that our findings represent a long-term average rather than a precise depiction of contemporary distribution. Consequently, our results should be interpreted as an overarching assessment of habitat relationships over time, providing a general understanding of ecological needs for the species.

## Data Availability

The data that support this study are available from Open PRAIRIE data repository at https://openprairie.sdstate.edu/nrm_datasets/17.

## References

[CIT0001] Aiello-Lammens ME , Boria RA, Radosavljevic A, Vilela B, Anderson RP, Bjornson R, Weston S. 2019. spThin: functions for spatial thinning of species occurrence records for use in ecological models. R package version 0.2.0. https://cran.r-project.org/package=spThin

[CIT0002] Allouche O , Tsoar A, Kadmon R. 2006. Assessing the accuracy of species distribution models: prevalence, kappa, and the true skills statistic (TSS). Journal of Applied Ecology 43(6):1223–1232. https://doi.org/10.1111/j.1365-2664.2006.01214.x

[CIT0003] Anderson MJ. 2001. Permutation tests for univariate or multivariate analysis of variance and regression. Canadian Journal of Fisheries and Aquatic Sciences 58(3):626–639. https://doi.org/10.1139/f01-004

[CIT0004] Araújo MB , New M. 2007. Ensemble forecasting of species distributions. Trends in Ecology & Evolution 22(1):42–47. https://doi.org/10.1016/j.tree.2006.09.010 17011070

[CIT0005] Aubry KB , Raley CM, McKelvey KS. 2017. The importance of data quality for generating reliable distribution models for rare, elusive, and cryptic species. PLoS One 12(6):e0179152. https://doi.org/10.1371/journal.pone.0179152 28640819 PMC5480872

[CIT0006] Balme GA , Hunter LTB, Slotow R. 2009. Evaluating methods for counting cryptic carnivores. Journal of Wildlife Management 73(3):433–441. https://doi.org/10.2193/2007-368

[CIT0007] Barbet-Massin M , Jiguet F, Albert CH, Thuiller W. 2012. Selecting pseudo-absences for species distribution models: how, where, and how many? Methods in Ecology and Evolution 3(2):327–338. https://doi.org/10.1111/j.2041-210X.2011.00172.x

[CIT0008] Barker WT , Whitman WC. 1988. Vegetation of the Northern Great Plains. Rangelands 10(6):266–272. http://hdl.handle.net/10150/640344

[CIT0009] Barnett KL , Facey SL. 2016. Grasslands, invertebrates, and precipitation: a review of the effects of climate change. Frontiers in Plant Science 7(2016):1196. https://doi.org/10.3389/fpls.2016.01196 27547213 PMC4974256

[CIT0010] Burcher CL , Valett HM, Benfield EF. 2007. The land-cover cascade: relationships coupling land and water. Ecology 88(1):228–242. https://doi.org/10.1890/0012-9658(2007)88[228:tlcrcl]2.0.co;2 17489471

[CIT0011] Cheeseman AE , Tanis BP, Finck EJ. 2021a. Temporal assessment of eastern spotted skunk geographic distribution. Southeastern Naturalist 20(sp11):24–38. https://doi.org/10.1656/058.020.0sp1104

[CIT0012] Cheeseman AE , Tanis BP, Finck EJ. 2021b. Quantifying temporal variation in dietary niche to reveal drivers of past population declines. Functional Ecology 35(4):930–941. https://doi.org/10.1111/1365-2435.13765

[CIT0013] Choate JR , Fleharty ED, Little RJ. 1973. Status of the spotted skunk, *Spilogale putorius*, in Kansas. Transactions of the Kansas Academy of Science 76(3):226–233. https://doi.org/10.2307/3627104

[CIT0014] Crabb WD. 1948. The ecology and management of the prairie spotted skunk in Iowa. Ecological Monographs 18(2):201–232. https://doi.org/10.2307/1948639

[CIT0015] Darwin C. 1859. On the origin of species by means of natural selection. London (UK): John Murray Publishing House.

[CIT0016] Dormann CF , Elith J, Bacher S, Buchmann C, Carl G, Carré G, García Marquéz JR, Gruber B, Lafourcade B, Leitão PJ, et al. 2013. Collinearity: a review of methods to deal with it and a simulation study evaluating their performance. Ecography 36(1):27–46. https://doi.org/10.1111/j.1600-0587.2012.07348.x

[CIT0018] Elith J , Graham CH, Anderson RP, Dudík M, Ferrier S, Guiśan A, Hijmans RJ, Huettmann F, Leathwick JR, Lehmann A, et al. 2006. Novel methods improve prediction of species’ distributions from occurrence data. Ecography 29(2):129–151. https://doi.org/10.1111/j.2006.0906-7590.04596.x

[CIT0019] ESRI. 2019. ArcMap. Version 10.7.1 [computer software]. Redlands (CA, USA): Environmental System Research Institute, Inc. http://www.esri.com

[CIT0021] Fick SE , Hijmans RJ. 2017. WorldClim 2: new 1-km spatial resolution climate surfaces for global land areas. International Journal of Climatology 37(12):4302–4315. https://doi.org/10.1002/joc.5086

[CIT0022] Fielding AH , Bell JF. 1997. A review of methods for the assessment of prediction errors in conservation presence/absence models. Environmental Conservation 24(1):38–49. https://doi.org/10.1017/s0376892997000088

[CIT0023] Fino S , Stafford JD, Pearse AT, Jenks JJ. 2019. Incidental captures of plains spotted skunks in Central South Dakota. Prairie Naturalist 51(1):33–36. digitalcommons.unl.edu

[CIT0024] Frankson R , Kunkel KE, Champion SM, Easterling DR, Umphlett NA, Stiles CJ. 2022. South Dakota State Climate Summary 2022. NOAA Technical Report NESDIS 150-SD. Silver Spring (MD, USA): NOAA/NESDIS; 5 pp.

[CIT0025] Gallant AJE , Karoly DJ, Gleason KL. 2014. Consistent trends in a modified climate extremes index in the United States, Europe, and Australia. Journal of Climate 27(4):1379–1394. https://doi.org/10.1175/jcli-d-12-00783.1

[CIT0026] GBIF.org. 2021. Home page. Copenhagen (Denmark): Global Biodiversity Information Facility. [accessed 13 Jan 2021]. https://www.gbif.org/

[CIT0027] Gompper ME. 2017. Range decline and landscape ecology of the eastern spotted skunk. In: MacDonald DW, Newman C, and Harrington LA, editors. Biology and conservation of Musteloids. Oxford (UK): Oxford University Press; p. 478–492.

[CIT0028] Gompper ME , Hackett HM. 2005. The long-term, range-wide decline of a once common carnivore: the eastern spotted skunk (*Spilogale putorius*). Animal Conservation 8(2):195–201. https://doi.org/10.1017/s1367943005001964

[CIT0029] Guisan, A, Thuiller W, Zimmerman NE. 2017. Habitat suitability and distribution models: with applications in R. Cambridge (UK): Cambridge University Press.

[CIT0030] Hanna E , Cooper TE, Hall RJ, Cappelen J. 2016. Greenland blocking index 1851–2015: a regional climate change signal. International Journal of Climatology 36(15):4847–4861. https://doi.org/10.1002/joc.4673

[CIT0031] Hesselbarth MHK. 2022. Landscapemetrics: landscape metrics for categorical map patterns. R package version 1.5.5. https://cran.r-project.org/package=landscapemetrics

[CIT0032] Hirzel AH , Lay GL, Helfer V, Randin C, Guisan A. 2006. Evaluating the ability of habitat suitability models to predict species presences. Ecological Modelling 199(2):142–152. https://doi.org/10.1016/j.ecolmodel.2006.05.017

[CIT0033] Kaskie KD , Wimberly MC, Bauman PJ. 2019. Rapid assessment of juniper distribution in prairie landscapes of the northern Great Plains. International Journal of Applied Earth Observation and Geoinformation 83(2019):101946. https://doi.org/10.1016/j.jag.2019.101946

[CIT0034] Kinlaw A. 1995. Spilogale putorius. Mammalian Species 511(511):1–7. https://doi.org/10.2307/0.511.1

[CIT0035] LaRose SH , MacPherson MP, Lesmeister DB, Hackett HM, Perry RW, Sasse DB, Gompper ME. 2022. Predicted distribution of plains spotted skunk in Arkansas and Missouri. Journal of Wildlife Management 86(2):e22165. https://doi.org/10.1002/jwmg.22165

[CIT0036] Lesmeister DB , Crowhurst RS, Millspaugh JJ, Gompper ME. 2013. Landscape ecology of eastern spotted skunk in habitats restored for red-cockaded woodpeckers. Restoration Ecology 21(2):267–275. https://doi.org/10.1111/j.1526-100X.2012.00880.x

[CIT0037] Lesmeister DB , Millspaugh JJ, Gompper ME, Mong TW. 2010. Eastern spotted skunk (*Spilogale putorius*) survival and cause-specific mortality in the Ouachita Mountains, Arkansas. American Midland Naturalist 164(1):52–60. https://doi.org/10.1674/0003-0031-164.1.52

[CIT0038] Lobo JM , Jiménez-Valverde A, Real R. 2007. AUC: a misleading measure of the performance of predictive distribution models. Global Ecology and Biogeography 17(2):145–151. https://doi.org/10.1111/j.1466-8238.2007.00358.x

[CIT0039] Loggins A , Shrader AM, Monadjem A, McCleery RA. 2019. Shrub cover homogenizes small mammals’ activity and perceived predation risk. Scientific Reports 9(1):16857. https://doi.org/10.1038/s41598-019-53071-y 31727923 PMC6856081

[CIT0040] Machar I , Harmacek J, Vrublova K, Filippovova J, Brus J. 2017. Biocontrol of common vole populations by avian predators *versus* rodenticide application. Polish Journal of Ecology 65(3):434–444. https://doi.org/10.3161/15052249pje2017.65.3.010

[CIT0041] Marmion M , Parvianen M, Luoto M, Heikkinen RK, Thuiller W. 2009. Evaluation of consensus methods in predictive species distribution modeling. Diversity and Distributions 15(1):59–69. https://doi.org/10.1111/j.1472-4642.2008.00491.x

[CIT0042] McDonough MM , Ferguson AW, Dowler RC, Gompper ME, Maldonado JE. 2022. Phylogenomic systematics of the spotted skunks (*Carnivora, Mephitidae, Spilogale*): additional species diversity and Pleistocene climate change as a major driver of diversification. Molecular Phylogenetics and Evolution 167(2022):107266. https://doi.org/10.1016/j.ympev.2021.107266 34302947

[CIT0043] Parmenter R , Yadav EP, Parmenter CA, Ettestad P, Gage KL. 1999. Incidence of plague associated with increased winter-spring precipitation in New Mexico. American Journal of Tropical Medicine and Hygiene 61(5):814–821. https://citeseerx.ist.psu.edu/document?repid=rep1&type=pdf&doi=c52e568fdc2e30cb92598535223edc2666a5a72c 10586917 10.4269/ajtmh.1999.61.814

[CIT0044] Perkins JC , Gibson AA, Wolaver BD, Labay BJ, Pierre JP, Dowler RC. 2022. An evaluation of detection methods for the plains spotted skunk. Wildlife Society Bulletin 46(5):e1376. https://doi.org/10.1002/wsb.1376

[CIT0045] Perkins JC III . 2017. Conservation status of the plains spotted skunk [master’s thesis]. [San Angelo (TX, USA)]: Angelo State University.

[CIT0046] Pimm SL , Jenkins CN, Brooks TM, Gittleman JL, Joppa LN, Raven PH, Roberts CM, Sexton JO. 2014. The biodiversity of species and their rates of extinction, distribution, and protection. Science 344(6187):1246752. https://doi.org/10.1126/science.1246752 24876501

[CIT0047] Polder E. 1968. Spotted skunk and weasel populations den and cover usage by northeast Iowa. Proceedings of the Iowa Academy of Science 75(1):142–145. https://scholarworks.uni.edu/pias

[CIT0048] Prugh LR , Stoner CJ, Epps CW, Bean WT, Ripple WJ, Laliberte AS, Brashares JS. 2009. The rise of the mesopredator. Bioscience 59(9):779–791. https://doi.org/10.1525/bio.2009.59.9.9

[CIT0049] R Core Team. 2023. R: a language and environment for statistical computing. Version 4.3.1. Vienna (Austria): R Foundation for Statistical Computing. www.R-project.org/

[CIT0050] Riitters KH , O’Neill RV, Jones KB. 1997. Assessing habitat suitability at multiple scales: a landscape-level approach. Biological Conservation 81(1–2):191–202. https://doi.org/10.1016/s0006-3207(96)00145-0

[CIT0051] Sofaer HR , Jarnevich CS, Pearse IS, Smyth RL, Auer S, Cook GL, Edwards TC, Guala GF, Howard TG, Morisette JT, et al. 2019. Development and delivery of species distribution models to inform decision-making. BioScience 69(7):544–557. https://doi.org/10.1093/biosci/biz045

[CIT0052] South Dakota Department of Transportation. 2020. Roadkill Location. DOT Wildlife Carcass Pickup. Created by SDDOT, using ArcMap 10.8.1, as a subset of the original dataset with Species=Small Animal.

[CIT0053] Swets JA. 1998. Measuring the accuracy of diagnostic systems. Science 240(4857):1285–1293. https://doi.org/10.1126/science.3287615 3287615

[CIT0054] Thuiller W , Georges D, Engler R, Breiner F. 2023. biomod2: ensemble platform for species distribution modeling. R package version 4.2-2. https://cran.r-project.org/package=biomod2

[CIT0055] Thuiller W , Guéguen M, Renaud J, Karger DN, Zimmermann NE. 2019. Uncertainty in ensembles of global biodiversity scenarios. Nature Communications 10(1):1–9. https://doi.org/10.1038/s41467-019-09519-w PMC6441032 30926936

[CIT0056] Tilman D , Clark M, Williams DR, Kimmel K, Polasky S, Packer C. 2017. Future threats to biodiversity and pathways to their prevention. Nature 546(7656):73–81. https://doi.org/10.1038/nature22900 28569796

[CIT0057] U.S. Census Bureau. 2020. South Dakota: 2020 census. www.census.gov

[CIT0058] U.S. Department of Agriculture [USDA] National Agricultural Statistics Service. 2017. Census of agriculture. www.nass.usda.gov/AgCensus

[CIT0067] U.S. Fish and Wildlife Service. 2012. Endangered and threatened wildlife and plants; 90-day finding on a petition to list the prairie gray fox, the plains spotted skunk, and a distinct population segment of the Mearns’ eastern cottontail in East-Central Illinois and western Indiana as endangered or threatened species. *Federal Register* 77(233):71759–71771. https://www.federalregister.gov/documents/2012/12/04/2012-29188/endangered-and-threatened-wildlife-and-plants-90-day-finding-on-a-petition-to-list-the-prairie-gray

[CIT0059] U.S. Fish and Wildlife Service. 2023. Proposed rules. Federal Register 88(181): 64870–64880. https://www.federalregister.gov/documents/2012/12/04/2012-29188/endangered-and-threatened-wildlife-and-plants-90-day-finding-on-a-petition-to-list-the-prairie-gray

[CIT0060] USDA. 2017. 2017 National Resources Inventory. Washington (DC, USA): Natural Resources Conservation Service. https://publicdashboards.dl.usda.gov/t/FPAC_PUB/views/RCADVPrimeFarmlandNRI20171/StatePrimeFarmland

[CIT0061] VertNet. 2016. Home page. [accessed 13 Jan 2021]. http://www.vertnet.org/

[CIT0062] Wei P , Lu Z, Song J. 2015. Variable importance analysis: a comprehensive review. Reliability Engineering and System Safety 142(2015):399–432. https://doi.org/10.1016/j.ress.2015.05.018

[CIT0063] White, K, Lonsinger RC, Stafford J. 2022. Distribution, habitat selection, and survival of plains spotted skunks in South Dakota, USA. Annual Performance Report to the South Dakota Department of Game, Fish and Parks. 18pp.

[CIT0064] Wilson GJ , Delahay RJ. 2001. A review of methods to estimate the abundance of terrestrial carnivores using field signs and observation. Wildlife Research 28(2):151–164. https://doi.org/10.1071/wr00033

[CIT0065] Wilson MC , Chen XY, Corlett RT, Didham RK, Ding P, Holt RD, Holyoak M, Hu G, Hughes AC, Jiang L, et al. 2016. Habitat fragmentation and biodiversity conservation: key findings and future challenges. Landscape Ecology 31(2016):219–227. https://doi.org/10.1007/s10980-015-0312-3

[CIT0066] Zarnetske PL , Edwards TC, Moisen GG. 2007. Habitat classification modeling with incomplete data: pushing the habitat envelope. Ecological Applications 17(6):1714–1726. https://doi.org/10.1890/06-1312.1 17913135

